# Endometrial Cancer Molecular Characterization: The Key to Identifying High-Risk Patients and Defining Guidelines for Clinical Decision-Making?

**DOI:** 10.3390/cancers13163988

**Published:** 2021-08-07

**Authors:** Regina Esi Mensimah Baiden-Amissah, Daniela Annibali, Sandra Tuyaerts, Frederic Amant

**Affiliations:** 1 Department of Oncology, Leuven Cancer Institute (LKI), KU Leuven (University of Leuven), 3000 Leuven, Belgium; reginaesimensimah.baidenamissah@kuleuven.be (R.E.M.B.-A.); daniela.annibali@kuleuven.be (D.A.); 2 Laboratory of Medical and Molecular Oncology (LMMO), Department of Medical Oncology, Vrije Universiteit Brussel (VUB), Universitair Ziekenhuis Brussel (UZ Brussel), 1090 Brussels, Belgium; Sandra.Tuyaerts@uzbrussel.be; 3 Centre for Gynecologic Oncology Amsterdam (CGOA), Antoni Van Leeuwenhoek-Netherlands Cancer Institute (Avl-NKI), University Medical Centre (UMC), 1066 CX Amsterdam, The Netherlands; 4 Department of Obstetrics and Gynecology, University Hospitals Leuven (UZ Leuven), 3000 Leuven, Belgium

**Keywords:** endometrial cancer, molecular risk stratification, targeted therapy, adjuvant therapy, genetic alterations, preclinical models

## Abstract

**Simple Summary:**

Endometrial carcinomas (EC) have been traditionally classified based on histopathology (types I and II). Determining the risk of a patient to experience disease recurrence is important to decide which patients need adjuvant treatment. The current endometrial carcinoma risk assessment approaches fail to accurately classify patients into low-and high-risk groups. Several studies suggest that combining molecular characteristics with the current risk classification in EC may improve patients’ stratification and treatment decision-making. In this review, we describe how evolving molecular trends can be used as prognostic factors to identify high-risk EC subpopulations. We also look at how the most recent patient-derived models can help researchers find new possible targets and treatments for EC patients.

**Abstract:**

Endometrial carcinomas (EC) are the sixth most common cancer in women worldwide and the most prevalent in the developed world. ECs have been historically sub-classified in two major groups, type I and type II, based primarily on histopathological characteristics. Notwithstanding the usefulness of such classification in the clinics, until now it failed to adequately stratify patients preoperatively into low- or high-risk groups. Pieces of evidence point to the fact that molecular features could also serve as a base for better patients’ risk stratification and treatment decision-making. The Cancer Genome Atlas (TCGA), back in 2013, redefined EC into four main molecular subgroups. Despite the high hopes that welcomed the possibility to incorporate molecular features into practice, currently they have not been systematically applied in the clinics. Here, we outline how the emerging molecular patterns can be used as prognostic factors together with tumor histopathology and grade, and how they can help to identify high-risk EC subpopulations for better risk stratification and treatment strategy improvement. Considering the importance of the use of preclinical models in translational research, we also discuss how the new patient-derived models can help in identifying novel potential targets and help in treatment decisions.

## 1. Introduction

### 1.1. Incidence and Symptoms

EC is the most commonly diagnosed malignancy in post-menopausal women. The highest incidence is seen between 55 and 64 years [1,2]. It is the sixth most frequent cancer in women worldwide and the most prevalent cause of mortality in the developed world, with 382,096 new cases and 89,929 deaths reported in 2018 [1,3,4]. The incidence of endometrial cancer differs throughout the world. Globally, North America and most of Europe have higher age-standardized incidence rates than the rest of the world [5,6,7]. The number of new cases of EC is expected to further increase, owing to the increasing aging population as well as the increase in obesity rates, as obesity is considered one of the main risk factors for EC [1,8]. Diabetes, obesity, nulliparity, early-onset menarche, late-onset menopause, and exposure to unopposed estrogens or tamoxifen are some of the well-known and major risk factors for EC development [8,9].

Early onset of signs and symptoms such as postmenopausal bleeding or spotting in 80% of patients leads to early diagnosis of endometrial cancer, where treatment efficacy is high. When compared to an advanced stage, these tumors are mostly diagnosed as FIGO (International Federation of Gynecology and Obstetrics) stage I or II, with a 5-year overall survival (OS) rate ranging from 74–97% [1,10,11]. Although the OS rates for advanced stage cases (FIGO stage III or IV) with possible distant metastasis differ from study to study, it is estimated that patients with stage III and IV have dismal 5-year OS rates, ranging from 57–66% and 20–26%, respectively, compared to early stage disease [10,11,12].

### 1.2. Classification

EC is traditionally divided into two main categories, type I and type II EC, which can be distinguished clinically, morphologically, histologically, and genetically. Type I ECs, commonly referred to as endometrioid EC (EECs), represent up to 80% of the newly diagnosed cases and account for 15–20% of recurrences. They are typically low-grade tumors associated with perimenopausal obese women. They are also hormone-dependent with a favorable prognosis (85% 5 years OS rate) [1,9].

Non-endometrioid ECs make up the majority of Type II ECs. They are more common in elderly women and are usually hormone-independent. This is a less common group (10–20%), but it is associated with a higher risk of disease recurrence and a poor prognosis (25–60% 5 years OS rate) [1,9]. The most frequent mutations in both type I and type II tumors are listed in Table 1 [6,10,13,14,15,16,17,18]. However, due to the heterogeneous nature of ECs, the possibility of having tumors containing both subtypes is high. This limits the application of a dualistic classification and hinders effective treatment, hence the need for additional markers to improve or complement the histological classification of ECs.

Phosphatase and tensin homolog (PTEN), Microsatellite instability (MSI), TP53 Tumor protein (TP53), Polymerase ɛ (POLE), Phosphatidylinositol-4,5-bisphosphate 3-kinase catalytic subunit alpha (PIK3CA), Phosphoinositide-3-kinase regulatory subunit 1 (PIK3R1), Catenin beta 1 (CTNNB1), AT-rich interaction domain 1A (ARID1A), KRAS proto-oncogene, GTPase (KRAS), Fibroblast growth factor receptor 2 (FGFR2), F-box and WD repeat domain containing protein 7 (FBXW7), Protein Phosphatase 2 scaffold subunit A alpha (PPP2R1A), Lysine Methyltransferase-2B (KMT2B), CCCTC-binding factor (CTCF), Chromodomain helicase DNA-binding protein 4 (CHD4), Ribosomal protein L22 (RPL22), AT-rich interaction domain 5B (ARID5B), CUB and Sushi multiple domains 3 (CSMD3), Collagen Type I Alpha 1 Chain (COL1A1), Human epidermal growth factor receptor 2 (HER2), Estrogen receptor (ER), progesterone receptor (PgR), Epithelial cadherin (E-cadherin), and Epidermal growth factor receptor (EGFR).

### 1.3. Current Treatment Options

In EC patients who are classified as low- or low-intermediate risk, surgery (abdominal or laparoscopic hysterectomy and bilateral salpingo-oophorectomy) is usually recommended [10,19,20]. Assigning adjuvant treatment to high-intermediate and high-risk subgroups depends on the presence of clinicopathological factors such as age, tumor grade, histologic features, the extent of myometrial invasion, and absence or presence of lymphovascular invasion [6,19,20]. Adjuvant radiotherapy–vaginal brachytherapy (VBT) and pelvic external beam radiotherapy (EBRT)—with or without adjuvant chemotherapy—are normally assigned to the high-intermediate risk and high-risk subgroups, respectively [21,22,23]. For advanced and recurrent endometrial cancers, the first-line treatment is chemotherapy (carboplatin and paclitaxel), which may be combined with hormonal therapy [24,25,26]. Although some clinical trials did show treatment benefits, other studies did not [27,28]. For localized recurrent disease surgery and radiation therapy (intensity-modulated radiation therapy (IMRT) or a combination of both) can be considered, while, for non-localized recurrent disease, progestin therapy (medroxyprogesterone acetate or megestrol acetate) is given [29]. For advanced EC patients with microsatellite instability-high (MSI-H)/mismatch repair deficiency (dMMR), the anti-PD-1 blockade, pembrolizumab, and Dostarlimab-gxly have been approved [30,31]. Furthermore, a combination of pembrolizumab and anti-VEGFR, lenvatinib was recently approved for treating patients with advanced EC with tumors that are not MSI-H or dMMR [32].

In this review, we will briefly discuss the four major EC molecular subtypes described by TCGA, their prognostic relevance, and future development. In addition, we will discuss the potential reclassification of high-risk ECs (HR-ECs) using molecular characterization for better patient stratification. Treatment benefit, clinical implication, and future trends in EC management based on molecular classification will also be summarized. We will also outline the importance of molecular characterization in pre-clinical models.

## 2. The Cancer Genome Atlas (TCGA) Molecular Classification for Endometrial Cancer

The use of the traditional risk assessment alone lacks the power to adequately preoperatively stratify patients into low- or high-risk groups for surgical planning and adjuvant treatment. This may result in over- and under-treatment of patients, which may have an impact on the results of various clinical trials because treatment is typically given to patients based on their risk categories. [33,34]. Integrating the clinicopathologic features with molecular biomarkers predictive of patient tumor behavior may provide more accurate risk stratification and improve patients’ prognosis [35,36,37].

The TCGA research network project used whole genome sequencing, exome sequencing, microsatellite instability (MSI), and copy number analysis in 373 EC cases [38]. The TCGA project identified four molecularly defined EC subgroups: (1) ultramutated/polymerase E (POLE) mutant—7%, (2) hypermutated/MSI–28%, (3) copy-number low/microsatellite stable (CNL)—39%, and (4) copy-number high/serous-like (CNH)–26%. After TCGA findings, several studies have confirmed the prognostic significance of the four molecular subgroups, with further advancement in terms of genetic alterations, especially in the CNL subgroup [34,37,38,39,40,41,42,43,44,45]. The description of the four main molecular subgroups of EC by TCGA with genetic mutations and associated prognostic significance are summarized in Table 2**.** However, their work had some limitations such as the use of: (1) expensive and impractical methods for routine clinical practice, and (2) fresh-frozen tumor samples, requiring special sample handling, making it difficult for every hospital to perform such analysis for every patient. To overcome these limitations, two research teams developed practical and cost-effective molecular classifiers to aid in better risk classification of EC. Firstly, the ProMisE (Proactive Molecular Risk Classifier for Endometrial Cancer) study used immunohistochemistry for mismatch repair proteins, TP53 Tumor protein (p53), and POLE as surrogates of molecular markers and risk-stratified a large cohort of young women (*n* = 319) of less than 50 years of age and defined prognosis in this cohort [34]. Secondly, Stelloo et al., to obtain a large series of high-risk ECs using inclusion criteria of the PORTEC3 (Postoperative Radiation Therapy in Endometrial Cancer 3) study, also confirmed the molecular classification of EC with further prognostic impact [37].

## 3. Post TCGA Molecular Classification and Prognostic Significance in Endometrial Cancer

### 3.1. Prognostic Impact of Histology and Grade

The TCGA molecular classification was performed on tumors from the two most frequent histological subtypes, endometrioid, and serous EC (307 endometrioid and 53 serous). To molecularly characterize other histotypes, other studies have proven that the four main molecular classifications in EC may be applied to, and may also be clinically relevant for, other rare histological subtypes such as undifferentiated/dedifferentiated, clear cell EC’s and carcinosarcomas [34,46,47,48]. This prompted further studies to better understand how the four molecular subgroups may influence the prognostic impact of clinicopathological parameters such as tumor grade and histology. According to the European Society for Medical Oncology (ESMO) guidelines for EC, FIGO stage I grade 1–2 (G1–2) EC are considered at low-to-intermediate risk, whereas grade 3 (G3) EC are considered at intermediate-high to high-risk [6].

In assessing the prevalence of the TCGA molecular subgroups across tumor grades, a systematic review and meta-analysis by Travaglino et al. in EEC reported that G1–2 EECs are predominantly in the CNL subgroup (63.5%), followed by the MSI subgroup (24.7%), the POLE-mt subgroup (6.2%), and the CNH subgroup (4.7%). G3 EECs were mostly seen in the MSI subgroup (39.7%), followed by the CNL subgroup (28%), the CNH subgroup (21.3%), and the POLE-mt subgroup (12.1%) [49].

In similar studies where both grade and histology across the four molecular subgroups were analyzed, it has been reported that most G1–2 associated with CNL (84.4%), followed by POLE-mt group (60.4%), the MSI group (52.6%) and lastly the CNH (10%). On the other hand, CNH constituted most of the G3 (90%), followed by MSI subgroup (47.4%), the POLE-mt group (39.6%), and then the CNL subgroup (15.6%) [50]. In terms of histotype distribution across the four molecular subgroups of EC, following TCGA, most CNL, MSI, and POLE-mt tumors were reported to be EEC, whereas CNH was categorized as serous/non-endometrioid EC with non-endometrioid carcinomas having a worse prognosis in each TCGA subgroup [38,50,51]. Importantly, they observed that the MSI subgroup has the highest risk of death and recurrence/progression of the disease, followed by the CNH subgroup, while non-endometrioid POLE-mt carcinomas showed variable prognosis. However, the EEC POLE-mt subgroup showed the best prognosis, with the CNH subgroup having the worst prognosis.

In applying molecular features in G3 EEC, four prognostic subgroups were identified. The 5-year recurrence-free survival (RFS) rates reported were as follows: POLE-mt 96%, MSI 77%, CNL 64%, and CNH 47% (*p* = 0.000001), respectively [41]. Combining molecular features with grade and histology in these studies indicates how some patients may be over or under-treated if only one of the risk assessment criteria (clinicopathological features or molecular features) is used [6]. For instance, the presence of grade 3 in the CNL group may prompt the use of adjuvant therapy, although the CNL subgroup is considered to have a good to intermediate prognosis. Again, even within G3, which is considered high-risk, when TCGA molecular characterization is applied, not all patients may need adjuvant therapy. Considering the fact that POLE-mt patients generally have a good prognosis, it would conceivably be more appropriate that patients with such mutations irrespective of grade and stage, but of endometrioid histology are considered low-risk and spared of unnecessary treatment to avoid over-treatment [41].

### 3.2. Prognostic Impact within High-Risk EC (HR-EC)

Classifying HR-EC histologically may be problematic since some of these tumors may show features of multiple histologic subtypes. However, their risk assessment is of utmost importance as this greatly influences treatment options. The ESMO-ESGO-ESTRO guidelines classification of ECs with a high risk of disease recurrence includes: (1) endometrioid (type I) FIGO stage IB grade 3 tumors (type I/G3ECs), (2) non-endometrioid tumors (type II), (3) advanced stages of any histological type, and (4) presence of lymphovascular space invasion (LVSI) [6,9]. In addition to the ESMO-ESGO-ESTRO risk classification of ECs, recently, a retrospective multicenter study within the European Network for Individualized Treatment of Endometrial Cancer (ENITEC) has identified a new risk category in ECs. Investigating the prognostic relevance of pre-operative immunohistochemical (IHC) biomarkers, the abnormal expression of p53/L1CAM/ER/PR was significantly associated with higher risk classification groups, which corresponded to the worst patient outcome within the high-risk subgroup [52]. Although HR-ECs are less prevalent, they are aggressive with a poor prognosis, and are therefore given adjuvant therapy [6,53,54].

Various predictive models have indicated the influence of molecular subtypes on prognostic relevance within HR-EC. [35,44,55,56,57,58]. In one study, favorable prognosis was reported in POLE-mt and MSI tumors in HR patients with 5 year recurrence-free survival (RFS) of 93% and 95%, compared to 42% and 52% for the CNH and the CNL group, respectively (*p* < 0.001) [39]. Léon-Castillo et al. [59], identified outcomes associated with molecular subgroups of patients with high-risk. They reported that patients classified as POLE-mt had the best RFS at 5 years of 98%, followed by 74% for those with CNL tumors, 72% for MSI tumors, and 48% among patients with CNH tumors (*p* < 0.001). The same trend was seen with OS at 5 years: 98% for POLE-mt, 88.5% CNL, 81.3% for MSI and 54.0% for CNH (*p* < 0.001). Regarding treatment outcome, the 5 year RFS with chemoradiation vs. radiotherapy alone was 59% vs. 36% (*p* = 0.019) for patients classified as CNH. The POLE-mt group did well regardless of treatment group; 100% vs. 97% (*p* = 0.637). Similar survival was seen in both treatment groups for MSI tumors; 68% vs. 76% (*p* = 0.428), and 80% vs. 68% (*p* = 0.243) for CNL [59].

Another group, using DNA damage response biomarkers to further reclassify the HR group, identified five prognostic subgroups within this population from best to worst prognosis: (1) group 1 “POLE mutated/Microsatellite unstable” > (2) group 2 “no specific molecular profile with no DNA damage” > (3) group 3 “TP53 mutated/Non-Homologous End-Joining negative” > (4) group 4 “no specific molecular profile with high DNA damage” > (5) group 5 “TP53 mutated/Non-Homologous End-Joining positive”; *p*  =  0.0002). They reported that, although the markers (δ-H2AX, RAD51, DNA-pk, FANCD2 and PARP-1) alone within the four molecular subgroups were not prognostic, δ-H2AX+ expression within CNL (Hazard Ratio  =  2.56; *p*  =  0.026) and DNA-pk+/FANCD2 within the CNH group (Hazard Ratio  =  4.95; *p*  =  0.009) significantly predicted poor disease free survival [60]. These results strongly highlight the prognostic value and impact of molecular classification in high-risk EC patients, advocating the need for the incorporation of molecular signatures in risk stratification for better refinement of treatment at the patient level. Apart from this, there are insufficient data on the prognostic value of surgical staging in the different molecular groups. For example, traditionally, lymph node status is the most important prognostic factor in EC. However, its importance in the different molecular subgroups to date has insufficiently been investigated. The same basically applies to peritoneal staging by omentectomy and/or peritoneal biopsies.

## 4. Treatment Benefit in HR-ECs under the Influence of TCGA Molecular Subtypes

Despite the disappointing outcomes of some clinical trials in HR-ECs [61,62,63], treatment benefit has been demonstrated in a subset of high-risk patients that are categorized in any of the four molecular subgroups. The outcome of immunotherapy treatment in the POLE-mt subgroup is very promising, due to their high mutational load, as evidenced by high numbers of tumor-infiltrating T cells [64,65]. In a sub-analysis of POLE-mt EC, where confirmed high-risk women were treated with VBT or EBRT, the 10 year RFS for the POLE-mt subgroup was 100% [66]. Due to the good prognosis in the POLE-mt subgroup, the high treatment response rates observed across multiple treatment regimens may imply that these tumors have some intrinsic characteristics (strong host immune response), independent of sensitivity to the treatment—hence the proposal by some authors to safely omit adjuvant therapy in early stage POLE-mt ECs [34,67]; however, further prospective supportive research is strongly needed before concluding on the use or omission of adjuvant therapy in the POLE-mt subgroup [68].

MSI tumors, like POLE-mt, are immunogenic, explaining the increased interest in immunotherapy in the MSI group [69,70,71]. For example, in a recent case report, two chemotherapy-resistant EC patients (one with MSI and the other with POLE-mt) showed an exceptional clinical response to the anti–PD-1, nivolumab [72]. Despite the negative prognostic markers associated with MSI [73,74], in a long-term study in high-intermediate risk (HIR) EC, MSI patients treated with VBT or EBRT had a 10-year cancer-specific survival (CSS) of 84.8%. Importantly, it was also reported that vaginal brachytherapy only was equally effective in preventing pelvic lymph node recurrences in the absence of prognostic markers such as p53abn, L1CAM, and substantial LVSI, indicating the possibility of low risk in the MSI subgroup [66].

In addition to approximately 90% mutation in the TP53 gene in CNH, overexpression of epidermal growth factor receptor 2 (HER2) has been observed in this subgroup [37]. In addition to treatment benefit that resulted from the proposal of adjuvant treatment escalation for both high-intermediate-risk and high-risk patients in CNH subgroup [59], other treatment benefits have been indicated. A recent randomized phase II clinical trial found that treating 61 patients with stages III and IV or recurrent HER2 positive serous EC with carboplatin-paclitaxel–trastuzumab reduced the risk of progression and increased PFS from 8 to 13 months when compared to carboplatin-paclitaxel alone. [75]. Again, trastuzumab alone or in combination with chemotherapy was found to be beneficial in serous EC with HER2 overexpression [76,77], though other studies using trastuzumab as a single agent failed to demonstrate any prognostic benefit [78,79].

The CNL subgroup, which lacks a distinct molecular pattern, is distinguished by a low mutational burden and is associated with a poor prognosis. Mutation of CTNNB1 is one of the most common molecular changes in this subgroup [41]. Although CTNNB1 exon 3 mutation is associated with decreased OS [44], patients with CTNNB1 mutations benefited from the combination of everolimus, letrozole, and metformin with acceptable toxicity [80], indicating that the CNL subgroup may benefit from treatments targeting glucose metabolism.

## 5. Potential Clinico-Molecular Guided Risk Stratification and Treatment Benefit in HR-ECs

Some retrospective studies revealed a potential impact of the molecular-based classification of EC on EC patients in the clinics. ESGO and ESTRO have recommended a new molecular-based clinicopathological risk stratification system for adjuvant treatment [81], where POLE-mt was assigned to the low-risk and MSI, CNH, and CNL were assigned to the intermediate-to high-risk group. In the high-risk group, chemoradiation and EBRT were recommended. In addition to the current clinico-molecular-based treatment recommendations in high-risk subgroups, it is thought that these patients could benefit from other treatment options based on their molecular profiles, as previously described in this review. To test these hypotheses, prospective randomized trials on patients stratified based on molecular subgroups should be conducted in the future.

The ongoing randomized PORTEC-4a trial (NCT03469674, ClinicalTrials.gov) is the first and only clinical trial that is investigating the use of an integrated clinicopathological and molecular risk profile for adjuvant therapy selection in stages I–II high-intermediate risk EC patients. [82]. Other prognostic factors, such as significant LVSI, L1CAM, and CTNNB1 mutation, are used in addition to the molecular profile to differentiate favorable, intermediate, and unfavorable profiles. The primary goal of this trial is to determine and compare the rates of vaginal recurrence after various post-surgery treatment options. Patients are randomized (2:1) to adjuvant treatment based on their molecular-integrated risk profile or standard adjuvant vaginal brachytherapy after surgery in this trial. Based on the molecular profile of the patients, 40% will receive brachytherapy, 5% will receive external beam radiation therapy, and 55% will be in the observation group.

In general, PORTEC trials lack data on prognosis based on tumor staging, either by lymphadenectomy or sentinel lymph node staging prior to EBRT administration. Nonetheless, the ongoing randomized PORTEC-4a clinical trial (NCT03469674, ClinicalTrials.gov) on molecular risk stratification may help guide adjuvant treatment decisions for better treatment strategy and patient management.

## 6. Potential of Molecular Targeted Therapies in HR ECs

Apart from histology, EC heterogeneity is reflected in genetic expression/alteration and other altered genetic pathways. HR-EC is associated with mutations in NRAS, APC, SMAD4, and expression of PD-L1/2, in addition to some of the genetic alterations listed in Table 1 and Table 2 [38,40,56,83,84,85]. Genetic mutations and molecular and signaling pathway abnormalities differ from patient to patient, emphasizing the need for personalized treatment. Based on genetic alterations, the benefit of targeted therapy in HR-ECs has been demonstrated.

Treatment response with the mTOR inhibitor temsirolimus in women with recurrent or metastatic EC, for example, has been tested in patients with mutated PTEN. In particular, 14% of 20 women who had not previously undergone chemotherapy showed a partial response and 69% had stable disease, whereas 4% of 25 women who had previously received chemotherapy showed a partial response and 48% had stable disease. When compared to single-agent chemotherapeutic or hormonal agent regimens, 17% of the patients experienced disease progression under temsirolimus [86].

In another study looking at the expression of ARID1A in various solid tumors, including EC, patients with ARID1A-mutated tumors had significantly longer median PFS after anti-PD-1/PD-L1 immunotherapy than patients with ARID1A wild-type tumors (11 months vs. 4 months, *p* = 0.006). In addition, multivariate analysis revealed that ARID1A alterations predicted longer PFS after the checkpoint blockade (HR (95% CI), 0.61 (0.39 to 0.94), *p* = 0.02) [87]. As a result, HR-ECs may benefit from a PD-1/PD-L1 checkpoint blockade followed by EZH2 inhibitors as a targeted therapy against ARID1A mutation.

**Figure 1:**
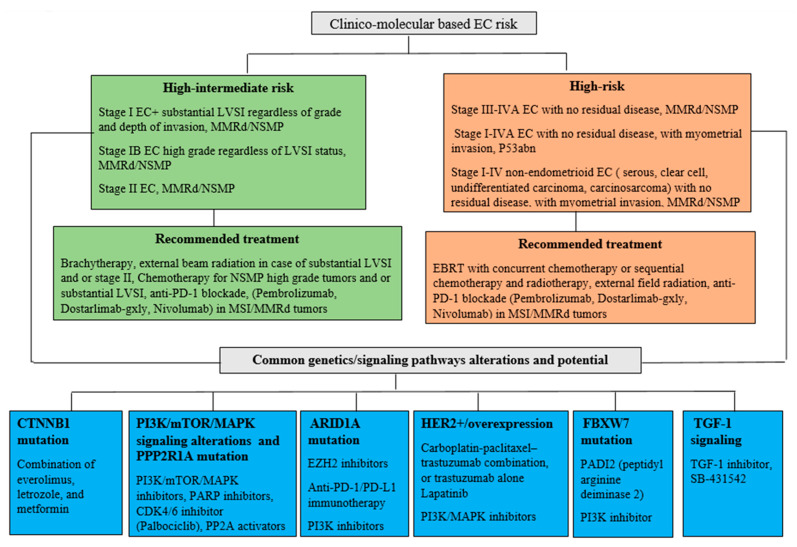
Molecular and genetic alterations in HR-EC with potential therapeutic targets and benefits. The classification of high-intermediate and high-risk EC based on the clinico-molecular risk stratification resulted in recommended treatment regimens in these categories. Anomalies in these tumors’ genetic and signaling pathways may also offer a novel treatment strategy and benefit based on specific alterations. Endometrial cancer (EC), lymphovascular space invasion (LVSI), mismatch repair deficiency (MMRd), non-specific molecular pattern (NSMP), catenin beta 1 (CTNNB1), mammalian target of rapamycin (mTOR), phosphoinositide 3-kinase (PI3K), microtubule associated protein kinase (MAPK), F-box and WD repeat domain containing protein 7 (FBXW7), protein phosphatase 2 scaffold subunit A alpha (PPP2R1A), human epidermal growth factor receptor 2 (HER2), AT-rich interaction domain 1A (ARID1A), poly adenosine diphosphate-ribose polymerase (PARP), enhancer of zeste homolog 2 (EZH2), transforming growth factor 1 (TGF-1), cyclin-dependent kinase 4/6 (CDK 4/6), and programmed cell death 1/ligand 1 (PD-1/PD-L1).

A modest clinical efficacy in EC patients with PD-L1 positive tumors treated with pembrolizumab has also been reported. In a phase Ib trial, 24 patients with PD-L1 positive recurrent metastatic EC were included in the study, and, after pembrolizumab treatment, the ORR was 13%. [88]. Figure 1 depicts a treatment decision in high-intermediate ECs under molecular/genetic influence.

## 7. Characterization of EC Patient-Derived Preclinical Models

The use of various preclinical models to enable potential drug target identification and also guide novel effective drug development is the pivot in EC research [89]. Patient-derived models include cell lines, explants, xenografts, and organoids. For more personalized patient care, these models can be molecularly characterized. Patient-derived models have several advantages, including the following: (1) they reflect several features of the original patient tumor specimen, (2) they can be used to identify potential genetic and molecular defects in genes, proteins, and signaling pathways in patients, and (3) they can predict the response of individual patients to different combinatorial treatment options, both conventional and novel anti-cancer therapeutics. Unlike lung, breast, and ovarian cancers, very few endometrial patient-derived cell lines have been generated and characterized [90,91,92,93]. Schrauwen et al. established and characterized for the first time seven primary EC cell lines and further developed subcutaneous and orthotopic xenograft models from some of the primary cell lines. Although the cell lines mimicked the genetic characteristics of the primary tumor, they observed that MSI status is the determinant of successfully establishing these cell lines [93].

Several studies in PDX and organoid models have demonstrated how these models, both histologically and genetically, resemble the primary tumor [93,94,95,96,97]. EC patient-derived preclinical models have contributed to novel findings in EC, which may influence the development of new anticancer drugs.

For example, Urick and Bell identified for the first time PADI2 (peptidyl arginine deiminase 2) as a novel therapeutic target for these tumors’ analysis of FBXW7 in serous EC cells [98]. In another study, inhibition of the mTOR and PI3K pathways in PTEN-deficient cell lines increased their sensitivity to PARP inhibitors, indicating the potential benefit of combining PI3K/mTOR inhibitors and PARP inhibitors in patients with PTEN mutations [99,100]. It has also been reported that TGF-1 signaling was associated with a high-risk recurrence phenotype in EC, implying that TGF-1 could be used to further stratify HR-ECs into high and low risk. TGF-1 inhibition with SB-431542, a specific TGF-1 inhibitor, slowed tumor growth and invasion, indicating a potential therapeutic benefit for HR-EC patients [101].

Depreeuw et al. established and characterized a panel of 24 EC PDX models. In one model derived from a patient who had previously received multiple lines of chemotherapy with PTEN, PIK3CA, and KRAS mutations, combining NVP-BEZ235 (dual PI3K-mTOR inhibitor) and AZD6244 (MEK1/2 inhibitor) treatment, resulted in stable disease [94]. Furthermore, they observed a significant enrichment of 2 molecular subgroups—MSI (62%) and POLE-mt (15%)—in the engrafted models, implying that immunotherapy may be beneficial in these models as well. A similar combinatorial effect of NVP-BEZ235 and AZD6244 was observed or confirmed in EC-derived primary cell lines, whereas NVP-BEZ235 alone was able to reduce tumor growth in vivo [102]. PTEN deficiency in EC PDX models also resulted in an anti-tumor effect with the CDK4/6 inhibitor, Palbociclib (PD-332991) [103].

A drug screening performed in EC patients-derived organoid cultures identified menin-MLL inhibitor (MI-136), as a potential therapeutic target for endometrial cancer due to its regulation of the HIF pathway [104]. Paclitaxel also synergized with an aldehyde dehydrogenase (ALDH) inhibitor to inhibit cancer proliferation in EC organoids with high ALDH levels [105].

There is no doubt that patient-derived preclinical EC models have paved the way for drug screening and novel drug development in ECs, but these models could be further improved. The way forward could be to molecularly risk-stratify these models into various risk categories and then test a treatment regimen based on their molecular profile. These preclinical findings will then be used to determine which drugs may perform better clinically or could be tested on humans, facilitating the design of novel clinical trials.

## 8. Conclusions and Future Perspectives

Treatment efficacy in EC, especially in the high-risk subgroup, has not yet shown satisfactory results, despite increased efforts in improving treatment strategy. Patient risk stratification is of utmost importance in assigning the most appropriate treatment regimen to avoid under- and or over-treatment of patients. The TCGA molecular classification of EC has added a risk assessment tool to the current risk stratification. In recent years, several retrospective studies after TCGA have indicated how incorporating molecular features or testing into traditional risk assessment may help in identifying the high-risk population, and further aid in reclassifying the HR-EC subgroup. Furthermore, the application of molecular or genetic risk profile in patient-derived EC preclinical models may facilitate the development and the evaluation of the efficacy of novel targeted drugs, as well as assist in identifying predictive/prognostic biomarkers to assess patient treatment response.

## Figures and Tables

**Table 1:** Common genetic/molecular alterations and frequencies in type I and type II EC.

Genetic Alteration	Type I (%)	Type II (%)	Reference
PTEN mutation	30–93.7	2–55	[13,14,15,16,17,18]
PTEN loss	80	5	[13,14,15,16,17,18]
MSI mutation	33–44	2–14	[13,14]
TP53 mutation	3–35	28–93	[13,15,16,17]
POLE mutation	11–20	2–7	[13]
PIK3CA mutation	22–90	10–50	[13,14,15,16,17]
PIK3R1 mutation	9–43	5–20	[13,17]
CTNNB1 mutation	19–52	2.7–4.8	[13,15]
ARID1A mutation	39–68.7	7–24	[6,10,18]
KRAS mutation	7–43	2–17	[13,14,15,16]
FGFR2 mutation	2–18	1–8	[13,16]
FBXW7 mutation	10–15	7.9–39	[13,15]
PPP2R1A mutation	2.5–13	7–43.2	[13,15,17]
KMT2B mutation	43.7	0	[17]
CTCF mutation	20.6	0	[17]
CHD4 mutation	0	16.3	[17]
RPL22 mutation	12	0	[18]
ARID5B mutation	11	0	[18]
CSMD3 mutation	0	11.6	[18]
COL1A1 mutation	0	11.6	[18]
HER2 overexpression	3–10	18–80	[14,16]
HER2 amplification	1–4	9–44	[13,16]
ER and PgR overexpression	70–73	19–24	[14]
E-cadherin loss	5–50	60–90	[16]
EGFR overexpression	46	34	[16]

**Table 2:** Description of TCGA EC molecular subgroups and prognosis.

Molecular Subgroup	Characteristics	Mutations	Somatic Mutation Rate	Prognosis
POLE-mt	EEC grade 3 tumors POLE exonuclease domain mutations, with V411L and P286R being the most common hotspot mutations in POLE	PTEN (94%), PIK3CA (71%), PIK3R1 (65%), FBXW7(82%), ARID1A (76%), KRAS (53), ARID5B (47%)	Very high (232 × 10^6^ mutations/Mb)	Excellent prognosis even in high grade tumors
MSI	EEC with high histological grade Most result of epigenetic silencing of MLH1 by promoter hypermethylation coupled with low PTEN levels and high phospho-AKT levels.	PTEN(88%), RPL22 (33%), KRAS (35%), PIK3CA (54%), PIK3R1 (40%), ARID 1A (37%)	High (18 × 10^6^ mutations/Mb)	Intermediate prognosis
CNH	Mainly serous endometrial tumors (94%) and mixed EC (62%), with fraction of EEC (12%), and about 25% of high-grade EEC. High copy number alterations.	TP53 (92%), PPP2R1A (22%), PIK3CA (47%) Recurrent amplifications in MYC, HER2, CCNE1, FGFR3 and SOX17	Low (2.3 × 10^6^ mutations/Mb)	Poor prognosis with significantly worse progression-free survival
CNL	Microsatellite stable tumors EEC (grades 1, 2 and 3) low somatic copy number alterations p53-wild-type (p53-wt) or few TP53 mutations	PTEN (77%), PIK3CA (53%), PIK3R1 (33%), ARID1A (42%), CTNNB1 (52%)	Low (2.9 × 10^6^ mutations/Mb)	Good-to-intermediate prognosis Significant prognostic impact Mutations in exon 3 of CTNNB1, L1CAM and ER/PR expression Amplification of chromosome 1q1q32.1

Polymerase mutant (POLE-mt), microsatellite instability (MSI), copy number high (CNH), copy number low (CNL), endometrioid endometrial cancer (EEC), mutL homolog 1 (MLH1), protein kinase B (AKT), phosphatase and tensin homolog (PTEN), TP53 Tumor protein (TP53), polymerase ɛ (POLE), phosphatidylinositol-4,5-bisphosphate 3-kinase catalytic subunit alpha (PIK3CA), phosphoinositide-3-kinase regulatory subunit 1 (PIK3R1), catenin beta 1 (CTNNB1), AT-rich interaction domain 1A (ARID1A), KRAS proto-oncogene, GTPase (KRAS), fibroblast growth factor receptor 3 (FGFR3), F-box and WD repeat domain containing protein 7 (FBXW7), protein phosphatase 2 scaffold subunit A alpha (PPP2R1A), ribosomal protein L22 (RPL22), AT-rich interaction domain 5B (ARID5B), human epidermal growth factor receptor 2 (HER2), cyclin E1 (CCNE1), MYC Proto-Oncogene (MYC), SRY-related HMG-box 17, and L1 cell adhesion molecule (L1CAM).

## Abbreviations

Akt	Protein kinase B
APC	Adenomatous polyposis coli
ARID1A	AT-rich interaction domain 1A
ARID5B	AT-rich interaction domain 5B
CDH1	Cadherin-1
CTCF	CCCTC-binding factor
CSS	Cancer specific survival
CTNNB1	Catenin beta 1
CCNE1	Cyclin E1
CDK4,6	Cyclin-dependent kinase 4,6
CHD4	Chromodomain helicase DNA-binding protein 4
COL1A1	Collagen Type I Alpha 1 Chain
CNH	Copy number high
CNL	Copy number low
CR	
CSMD3	CUB and Sushi multiple domains 3
DNA-PK	DNA-dependent protein kinase
EC	Endometrial cancer
EEC	Endometrioid endometrial cancer
EZH2	Enhancer of zeste homolog 2
ER/PR	Estrogen/progesterone receptor
EGFR	Epidermal growth factor receptor
E-cadherin	Epithelial cadherin
EBRT	External beam radiotherapy
ENITEC	European Network for Individualized Treatment of Endometrial Cancer
ESGO	European Society of Gynaecological Oncology
ESMO	European Society for Medical Oncology
ESTRO	European SocieTy for Radiotherapy & Oncology
FANCD2	Fanconi Anemia, Complementation Group D2
FBXW7	F-box and WD repeat domain containing protein 7
FGFR2	Fibroblast growth factor receptor 2
GOG	Gynecologic Oncology Group
δ-H2AX	H2A histone family member X
HER2	Human epidermal growth factor receptor 2
HIR	High–intermediate risk
HRD	Homologous recombination deficiency
HR-ECs	High-risk endometrial cancer
IMRT	Intensity-modulated radiation therapy
FIGO	International Federation of Gynecology and Obstetrics
KRAS	KRAS proto-oncogene, GTPase
L1CAM	L1 cell adhesion molecule
KMT2B	Lysine Methyltransferase-2B
LVSI	Lymphovascular space invasion
mTOR	Mammalian target of rapamycin
MSI	Microsatellite instability
MSI-H	Microsatellite instability-high
MMR	Mismatch repair
SMAD4	Mothers against decapentaplegic homolog 4
MLH	MutL homolog 1
MYC	MYC Proto-Oncogene
NRAS	Neuroblastoma RAS viral oncogene homolog
OS	Overall survival
ORR	Overall response rate
PADI2	Peptidyl arginine deiminase 2
PARP1	PARP Poly (ADP-ribose) polymerase 1
PD-1	Programmed cell death protein 1
PD-L1/2	Programmed cell death protein ligand ½
PI3K	Phosphoinositide 3-kinase
PIK3CA	Phosphatidylinositol-4,5-bisphosphate 3-kinase catalytic subunit alpha
PIK3R1	Phosphoinositide-3-kinase regulatory subunit 1
PDX	Patient-derived xenograft
POLE	Polymerase ɛ
POLE-mt	Polymerase mutant
PORTEC3	Postoperative Radiation Therapy in Endometrial Cancer 3
PFS	Progression free survival
ProMisE	Proactive Molecular Risk Classifier for Endometrial Cancer
PPP2R1A	Protein Phosphatase 2 scaffold subunit A alpha
PgR	Progesterone receptor
PR	Partial response
PTEN	Phosphatase and tensin homolog
RAD51	RAD51 recombinase
RFS	Recurrence-free survival
RPL22	Ribosomal protein L22
SOX17	SRY-related HMG-box 17
p53	TP53 Tumor protein
TCGA	The Cancer Genome Atlas
TGF-β1	Transforming growth factor beta 1
VBT	Vaginal brachytherapy
VEGFR	Vascular endothelial growth factor receptor
Wnt	Wingless/integration-1
